# Adherence to protective measures among healthcare workers in the UK: a cross-sectional study

**DOI:** 10.1136/emermed-2021-211454

**Published:** 2021-11-30

**Authors:** Louise E Smith, Danai Serfioti, Dale Weston, Neil Greenberg, G James Rubin

**Affiliations:** 1 Institute of Psychiatry Psychology & Neuroscience, King's College London, London, UK; 2 School of Psychology, University of Derby, Derby, UK; 3 Behavioural Science Team, UK Health Security Agency, London, UK

**Keywords:** COVID-19, global health, infectious diseases, disaster planning and response, first responders

## Abstract

**Background:**

Healthcare workers (HCWs) are frontline responders to emergency infectious disease outbreaks such as COVID-19. To avoid the rapid spread of disease, adherence to protective measures is paramount. We investigated rates of correct use of personal protective equipment (PPE), hand hygiene and physical distancing in UK HCWs who had been to their workplace at the start of the COVID-19 pandemic and factors associated with adherence.

**Methods:**

We used an online cross-sectional survey of 1035 UK healthcare professionals (data collected 12–16 June 2020). We excluded those who had not been to their workplace in the previous 6 weeks, leaving us with a sample size of 831. Respondents were asked about their use of PPE, hand hygiene and physical distancing in the workplace. Frequency of uptake was reported descriptively; adjusted logistic regressions were used to separately investigate factors associated with adherence to use of PPE, maintaining good hand hygiene and physical distancing from colleagues.

**Results:**

Adherence to personal protective measures was suboptimal (PPE use: 80.0%, 95% CI 77.3 to 82.8; hand hygiene: 67.8%, 95% CI 64.6 to 71.0; coming into close contact with colleagues: 74.7%, 95% CI 71.7 to 77.7). Adherence to PPE use was associated with having received training about health and safety in the workplace for COVID-19, greater perceived social pressure to adopt the behaviour and availability of PPE. Non-adherence was associated with fatalism about COVID-19 and greater perceived difficulty of adopting protective measures. Workplace design using markings to facilitate distancing was associated with adherence to physical distancing.

**Conclusions:**

Uptake of personal protective behaviours among UK HCWs at the start of the pandemic was variable. Factors associated with adherence provide insight into ways to support HCWs to adopt personal protective behaviours, such as ensuring that adequate PPE is available and designing workplaces to facilitate physical distancing.

**Key messages**

**What is already known on this subject**

- Two rapid reviews have identified rates of, and factors associated with, adherence to use of personal protective equipment (PPE) among healthcare workers, but most studies included investigated infectious diseases other than COVID-19 (eg, H1N1 pandemic influenza, seasonal influenza, severe acute respiratory syndrome, Middle East respiratory syndrome or tuberculosis).
- Data investigating rates of uptake and factors associated with hand hygiene and physical distancing among healthcare workers during infectious disease outbreaks are also lacking.
- Rates of adherence to personal protective behaviours among healthcare workers in the UK at the start of the COVID-19 pandemic and factors associated with adherence are unknown.

**What this study adds**

- In this survey of UK healthcare workers, adherence to personal protective measures was variable (PPE use: 80.0%; hand hygiene: 67.8%; physical distancing: 25.3%) among those who had been to their place of work in the last 6 weeks (n=831).
- Adherence to protective measures was associated with having received health and safety training and perceiving social pressure to adopt the behaviour. Greater perceived safety from COVID-19 in the workplace was also associated with adherence. Non-adherence was associated with fatalism for catching COVID-19 and greater perceived difficulty of adopting protective measures.
- Training that targets factors associated with adherence, clear environmental cues and promoting an organisational culture of adherence may help improve adherence to personal protective behaviours in healthcare workers.

## Introduction

To mitigate the spread of COVID-19, protective measures have been recommended in health and social care settings. These include use of personal protective equipment (PPE), good hand hygiene and physical distancing.^1–3^ However, these measures are not effective if healthcare workers (HCWs) do not or cannot adhere to them. There are few studies investigating uptake of personal protective behaviours (PPBs) among HCWs. Most available literature is from before the COVID-19 pandemic and does not report rates of uptake, instead reviewing factors associated with uptake.^4–6^ One systematic review has estimated median compliance rates to hand hygiene in hospitals at 40%, although this review is now outdated, including studies published before 1 January 2009.^7^ The COVID-19 pandemic has led to an intense and sustained information campaign across the whole of society aimed at improving rates of wearing a face covering in many settings and maintaining good hand hygiene. This has been accompanied by more specific workplace campaigns targeted at HCWs. It is unknown whether this has influenced rates of uptake of protective behaviours or whether factors previously identified as being associated with uptake of protective behaviours among HCWs remain relevant given the society-wide changes that have been seen.

We conducted a cross-sectional survey of UK healthcare professionals at the start of the COVID-19 pandemic to determine their use of PPE, good hand hygiene and physical distancing in the workplace, and to investigate factors associated with adherence.

## Method

We commissioned the market research company YouGov to carry out this cross-sectional survey, between 12 and 16 June 2020.

**Figure 1:**
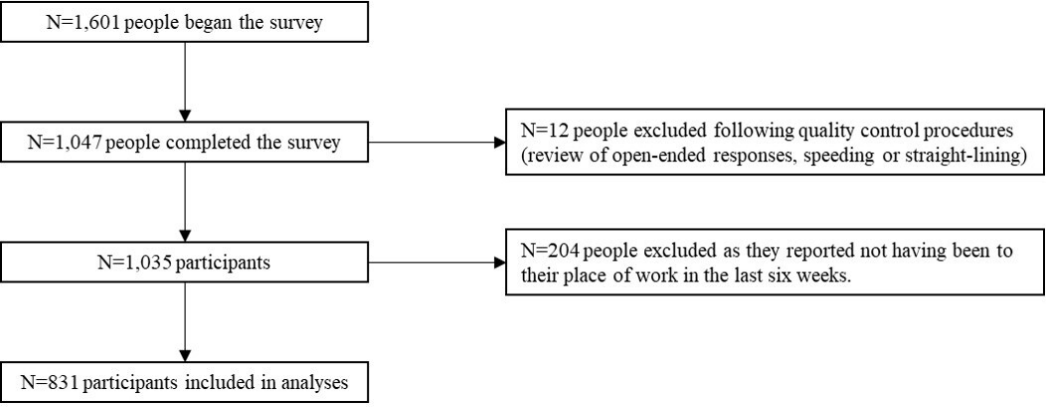
Flow chart of participants.

Participants were recruited from YouGov’s online research panel (n=800 000+ UK adults) and were eligible if they were 18 years or older, lived in the UK and worked in the healthcare sector (self-reported) (figure 1). For this study, we excluded participants who reported that they had not been to their place of work in the last 6 weeks. Quota sampling was used, based on occupational group with targets set to reflect the NHS staff survey. Through an automated sampling process, YouGov’s survey management software sets controls so that the respondents who are in a quota that has already been met are prevented from taking part. Participants were reimbursed in points (equivalent to approximately 50 p) redeemable as cash, gift vouchers or charitable donations.

### Study materials

We carried out telephone interviews with five clinical and administrative staff working in healthcare settings to inform survey questions. Full quantitative survey questions are available in the supplementary materials. Participants were only invited to complete survey materials if they had previously reported to the market research company that they worked in the healthcare sector.

We asked participants about their use of PPE (mask, gloves, apron or gown and face or eye protection); hand washing behaviour; and whether they had been in close contact with a colleague (within 2 m for 15 min or more or direct physical contact) the most recent time they were at work.

Participants were asked about their workplace environment; perceived risk of COVID-19; whether they had had COVID-19; perceived effectiveness of PPBs; perceived social pressure to adopt PPBs (thinking colleagues took PPBs seriously and would notice if you did not adopt them); and perceived safety from COVID-19. We also asked participants about how credible they perceived information from the NHS about PPE to be using an adapted form of the Meyer Credibility Index.^8^

Participants also reported their personal and occupational characteristics and whether they or a member of their household had recently experienced COVID-19 symptoms.

### Analysis

We aimed to recruit 1000 HCWs to give a 95% CI of plus or minus 3% for the prevalence estimate for each survey item.

Descriptive statistics are reported as means and SDs (continuous data) or frequencies and percentages (categorical data).

A series of logistic regressions determined univariable associations for: (A) total adherence to the use of PPE, (B) hand washing when needed at work and (C) close contact with colleagues at work. We investigated associations with personal and occupational characteristics, work environment and psychological and situational factors. A second set of logistic regressions determined multivariable associations for between our three outcomes and personal and occupational characteristics, work environment, and psychological and situational factors, controlling for personal and occupational characteristics (sex, age, region of place of work, sector, work setting and face-to-face contact with patients or service users).

Data were weighted by occupation group in the NHS workforce.

Due to the large number of analyses run on each outcome (up to 30), we applied a Bonferroni correction to our results (p≤0.002). Only results of adjusted analyses reaching significance are reported narratively. Unadjusted results are reported in tables; results not reaching significance are reported in the supplementary materials.

## Results

Among the 1035 HCWs who responded, 831 had been to their workplace in the previous 6 weeks. Most study participants were female, worked in the public sector and worked in a clinical setting (table 1).

**Table 1:** Participant characteristics

Characteristic	% (N)
Sex	
Male	28.1 (233)
Female	71.9 (598)
Age	
18–34 years	10.5 (87)
35–44 years	21.0 (174)
45–54 years	30.6 (255)
55+ years	37.9 (315)
Region of place of work	
North East	4.7 (39)
North West	12.3 (102)
Yorkshire and the Humber	8.2 (68)
East Midlands	6.8 (57)
West Midlands	7.4 (62)
East of England	6.3 (53)
London	8.3 (69)
South East	13.6 (113)
South West	10.9 (90)
Wales	6.2 (52)
Scotland	13.1 (109)
Northern Ireland	2.2 (18)
Sector	
Private	22.7 (189)
Public	77.3 (643)
Occupational group	
Allied health professionals/healthcare scientists/scientific and technical, public health/health improvement, commissioning managers/support staff, wider healthcare team (including admin and clerical, HR, finance, IT, facilities and maintenance), general management and other occupational group	50.6 (326)
Medical and dental, ambulance, registered nurses and midwives, nursing or healthcare assistants and social care	49.4 (317)
Work setting	
Pharmacy, dentist, opticians, clinical commissioning group, mental health trust/service, community services, local authority, school, university and other	27.4 (228)
NHS hospital, private hospital/ clinic, General Practice (GP) surgery/health centre, walk-in centre, ambulance trust/service and care home	72.6 (603)
Face-to-face contact with patients/service users	
No	17.3 (144)
Yes, occasionally	15.7 (131)_
Yes, frequently	67.0 (557)
Frequency of contact with patients with COVID-19 or staff who worked closely with patients with COVID-19	
I am never in contact myself with patients who have COVID-19 or anyone who has regular contact with patients who have COVID-19	34.0 (282)
I am never in contact myself with patients who have COVID-19 but work closely with staff who have regular contact with patients who have COVID-19	16.5 (137)
I am rarely in contact myself with patients who have COVID-19	18.8 (156)
I am sometimes in contact myself with patients who have COVID-19	20.4 (170)
I am often in contact myself with patients who have COVID-19	10.3 (86)
Had, or currently have, COVID-19	
Think have not had COVID-19 and do not have it now	78.2 (567)
Think have had COVID-19 or have it now	21.8 (158)
Symptoms of COVID-19 in household	
None present	96.5 (790)
Present	3.5 (28)
PPE	
Did not completely adhere to use of PPE	20.0 (166)
Completely adherent to use of PPE	80.0 (665)
Hand hygiene	
Did not wash their hands every time needed	32.2 (268)
Washed their hands every time needed	67.8 (564)
Physical distancing	
Were not in close contact with colleagues in the workplace	25.3 (210)
Were in close contact with colleagues in the workplace	74.7 (621)

PPE, personal protective equipment.

### Personal protective equipment

Among participants, 80.0% (n=665, 95% CI 77.3 to 82.8) reported completely adhering to use of PPE the most recent time they were at work. Factors independently associated with complete adherence were older age; having been given all the correct PPE needed to do one’s job; having enough information about what PPE to use and when to use it; receiving adequate health and safety training at work during the COVID-19 pandemic; thinking that colleagues take PPE and social distancing seriously; and feeling safe from COVID-19 at work (table 2).

**Table 2:** Factors associated with adherence to use of PPE in the workplace

Participant characteristics	Did not completely adhere to use of PPE n=166, n (%)	Completely adherent to use of PPE n=665, n (%)	OR (95% CI) for complete adherence to use of PPE	Adjusted OR (95% CI)* for complete adherence to use of PPE
Age				
18–34 years	31 (35.6)	56 (64.4)	Reference	Reference
35–44 years	39 (22.4)	135 (77.6)	1.94 (1.11 to 3.41)	1.69 (0.92 to 3.09)
45–54 years	46 (18.0)	209 (82.0)	2.55 (1.49 to 4.39)†	2.00 (1.12 to 3.58)
55 years and over	49 (15.6)	266 (84.4)	3.03 (1.78 to 5.16)†	2.64 (1.49 to 4.66)†
Face-to-face contact with patients/service users ‡				
No	4 (2.8)	140 (97.2)	Reference	Reference
Yes, occasionally	13 (9.9)	118 (90.1)	0.25 (0.08 to 0.80)	0.25 (0.08 to 0.81)
Yes, frequently	150 (26.9)	407 (73.1)	0.07 (0.03 to 0.21)†	0.07 (0.02 to 0.21)†
Frequency of contact with patients with COVID-19 or staff who worked closely with patients with COVID-19 ‡				
I am never in contact myself with patients who have COVID-19 or anyone who has regular contact with patients who have COVID-19	29 (10.3)	253 (89.7)	Reference	Reference
I am never in contact myself with patients who have COVID-19 but work closely with staff who have regular contact with patients who have COVID-19	21 (15.3)	116 (84.7)	0.63 (0.34 to 1.15)	0.37 (0.19 to 0.74)
I am rarely in contact myself with patients who have COVID-19	44 (28.0)	113 (72.0)	0.30 (0.18 to 0.50)†	0.36 (0.20 to 0.63)†
I am sometimes in contact myself with patients who have COVID-19	49 (28.8)	113 (72.0)	0.28 (0.17 to 0.47)†	0.43 (0.25 to 0.77)
I am often in contact myself with patients who have COVID-19	23 (27.1)	62 (72.9)	0.31 (0.17 to 0.57)†	0.55 (0.28 to 1.10)
I have received adequate training in my workplace for the purposes of health and safety during the COVID-19 pandemic (ie, correct use of PPE and social distancing), mean (±SD)§	3.30 (±1.22)	3.59 (±1.16)	1.22 (1.06 to 1.41)	1.33 (1.14 to 1.55)†
I am given all the correct PPE that I need to do my job safely, mean (±SD)§	3.45 (±1.20)	3.82 (±1.06)	1.34 (1.15 to 1.55)†	1.38 (1.18 to 1.63)†
I have enough information about which PPE to use and when to use it, mean (±SD)§	3.82 (±1.02)	4.03 (±0.93)	1.24 (1.05 to 1.47)	1.37 (1.13 to 1.66)†
It does not really matter what I do, I will probably catch COVID-19 anyway, mean (±SD)§	2.84 (±1.00)	2.48 (±0.95)	0.68 (0.58 to 0.81)†	0.71 (0.59 to 0.86)†
I am angry about the way PPE has been given out to me or other HCWs, mean (±SD)§	3.31 (±1.30)	2.93 (±1.24)	0.79 (0.69 to 0.90)†	0.78 (0.68 to 0.91)†
I feel safe from COVID-19 at work, mean (±SD)§	2.69 (±1.01)	3.12 (±1.05)	1.47 (1.25 to 1.74)†	1.50 (1.25 to 1.80)†
There is no point bothering with PPE around colleagues or social distancing if you already have a lot of contact with COVID-19 patients, mean (±SD)§	2.21 (±1.03)	1.83 (±0.88)	0.66 (0.56 to 0.79)†	0.67 (0.56 to 0.81)†
My colleagues seem to take PPE and social distancing seriously, mean (±SD)§	3.36 (±1.12)	3.75 (±1.02)	1.41 (1.20 to 1.65)†	1.48 (1.24 to 1.77)†
Wearing PPE makes it hard for me to do my job properly, mean (±SD)§	3.68 (±1.05)	3.11 (±1.09)	0.61 (0.51 to 0.72)†	0.64 (0.54 to 0.77)†

For continuous variables, where N is the same as the column heading, it is not reported in individual cells.

*Adjusting for sex, age, region of place of work, sector, work setting and face-to-face contact with patients or service users.

†P≤0.002 (applying Bonferroni correction).

‡The number of valid cases in the table is different from the total count due to the use of weighted data and rounding errors.

§Five-point scale: 1=strongly disagree to 5=strongly agree.

HCWs, healthcare workers; PPE, personal protective equipment.

Complete adherence was reported most frequently among participants who stated that they were never in contact with patients with COVID-19 or with staff who had close contact with patients who had COVID-19 (89. 7%). Poorer adherence was significantly associated with ‘often’, ‘sometimes’ or ‘rarely’ being in contact with patients with COVID-19. Poorer adherence was reported by those agreeing that wearing PPE makes it difficult to do one’s job; thinking there is no point bothering with PPE or social distancing if you have a lot of contact with patients with COVID-19; thinking that you will probably catch COVID-19 anyway no matter what you do; and being angry about the way PPE had been given out to you or other HCWs (table 2).

### Hand hygiene

Two-thirds of participants (67.8%, n=564, 95% CI 64.6 to 71.0) reported washing their hands ‘every time [they] needed to’ the most recent time they were at work. No personal, environmental, psychological or situational factors were significantly associated with hand hygiene (online supplemental materials).

10.1136/emermed-2021-211454.supp1 Supplementary data

10.1136/emermed-2021-211454.supp2 Supplementary data

10.1136/emermed-2021-211454.supp3 Supplementary data

### Physical distancing

Three-quarters of participants (74.7%, n=621, 95% CI 71.7 to 77.7) reported having come into close contact with a colleague the most recent time they were at work. Factors independently associated with this outcome were working in the public sector; greater perceived difficulty of physical distancing in the workplace; thinking that there is no point bothering with PPE or social distancing if you have a lot of contact with patients with COVID-19; and being aware of others in your workplace who had been seriously ill from COVID-19 (table 3).

**Table 3:** Factors associated with close contact in the workplace

Participant characteristics	Were not in close contact with colleagues in the workplace (n=210)	Were in close contact with colleagues in the workplace (n=621)	OR (95% CI) for being in close contact with a colleague	Adjusted OR (95% CI)* for being in close contact with a colleague
Sector †				
Private	74 (39.2)	115 (60.8)	Reference	Reference
Public	137 (21.3)	506 (78.7)	2.39 (1.69 to 3.38)‡	2.43 (1.66 to 3.56)‡
My workplace has clear markings which help me stay 2 m away from other people, mean (±SD)§	3.10 (±1.34)	2.51 (±1.24)	0.70 (0.62 to 0.79)‡	0.71 (0.62 to 0.80)‡
I have received adequate training in my workplace for the purposes of health and safety during the COVID-19 pandemic (ie, correct use of PPE and social distancing), mean (±SD)§	3.88 (±1.05)	3.42 (±1.20)	0.69 (0.60 to 0.80)‡	0.69 (0.59 to 0.81)‡
The way my workplace is designed makes it easy for me to stay 2 m away from other people, mean (±SD)§	3.09 (±1.23)	2.04 (±1.08)	0.49 (0.42 to 0.56)‡	0.49 (0.42 to 0.57)‡
Perceived ease of physical distancing in the workplace, mean (±SD) (range 0 (most easy) to 30 (most difficult))	12.68 (±5.99)	18.28 (±5.99)	1.16 (1.13 to 1.20)‡	1.16 (1.12 to 1.20)‡
Perceived credibility of information from the NHS about PPE, mean (±SD) (range 4 (lowest) to 20 (highest))	13.44 (±2.20)(n=185)	12.61 (±2.34)(n=558)	0.85 (0.79 to 0.92)‡	0.88 (0.82 to 0.95)‡
As far as I’m aware, there are people from my workplace who have been seriously ill with COVID-19, mean (±SD)§	2.91 (±1.47)	3.45 (±1.32)	1.33 (1.19 to 1.49)‡	1.22 (1.07 to 1.38)‡
I feel safe from COVID-19 at work, mean (±SD)§	3.37 (±0.97)	2.92 (±1.06)	0.65 (0.55 to 0.76)‡	0.67 (0.57 to 0.80)‡
There is no point bothering with PPE around colleagues or social distancing if you already have a lot of contact with COVID-19 patients, mean (±SD)§	1.65 (±0.82)	1.99 (±0.94)	1.59 (1.31 to 1.93)‡	1.52 (1.24 to 1.87)‡
Social distancing around colleagues at work is an effective way to protect against COVID-19, mean (±SD)§	4.11 (±0.75)	3.68 (±0.89)	0.52 (0.42 to 0.65)‡	0.56 (0.45 to 0.69)‡
If I don’t maintain social distancing at work, my colleagues will notice, mean (±SD)§	3.92 (±0.96)	3.31 (±1.08)	0.55 (0.46 to 0.65)‡	0.57 (0.47 to 0.68)‡

For continuous variables, where N is the same as the column heading, it is not reported in individual cells.

*Adjusting for sex, age, region of place of work, sector, work setting and face-to-face contact with patients or service users.

†The number of valid cases in the table is different from the total count due to the use of weighted data and rounding errors.

‡P≤0.002 (applying Bonferroni correction).

§Five-point scale: 1=strongly disagree to 5=strongly agree.

PPE, personal protective equipment.

Close contact was less likely to be reported among those who stated their workplace was designed to make it easy for them to stay 2 m away from other people; they had received adequate health and safety training at work during the COVID-19 pandemic; their workplace had clear markings to help them stay 2 m away from other people; thinking that social distancing around colleagues at work was an effective way of preventing the spread of COVID-19; that their colleagues would notice if they did not maintain social distancing; feeling safe from COVID-19 at work; and perceiving information from the NHS about PPE to be more credible (table 3).

## Discussion

Adherence to PPBs among HCWs in the first wave of the COVID-19 pandemic was imperfect. Given our use of self-report measures, these estimates of adherence are likely overestimates. Factors associated with complete adherence to PPE and physical distancing included having received training about health and safety in the workplace for COVID-19 and greater perceived social pressure to adopt protective behaviours. Non-adherence was associated with thinking there was ‘no point’ bothering with PPE or social distancing if you had a lot of contact with patients with COVID-19 (fatalism) and greater perceived difficulty of using the measures (including thinking PPBs made it difficult to do your job). Availability of PPE, workplace design to facilitate distancing and greater perceived information sufficiency were also associated with adopting individual PPBs. Factors associated with adoption of PPBs in our study were similar to those identified by two recent rapid reviews of HCW adherence to infection control measures in which most studies were conducted on infectious disease outbreaks other than COVID-19. These reviews also found that wearing PPE was associated with having an organisational culture that encourages adherence, while non-adherence was associated with shortages of PPE, inadequate guidance, perceived negative impact of adhering (eg, impairing ability to communicate with patients) and seeing other colleagues not adhering to PPE.^4 5^

Contrary to previous findings,^5^ participants who reported more patient contact were less likely to fully adhere to use of PPE. This may be a function of the greater number of times that PPE was necessary—allowing more opportunities for non-adherence. While not previously investigated with reference to PPBs in HCWs, we found that anger about how PPE had been distributed was associated with incomplete adherence. Participants who were angrier about distribution of PPE may have had more patient contact and been more fatalistic about COVID-19, themselves both directly associated with reduced adherence. However, this post hoc explanation is speculative and should be taken with caution.

This study has several limitations. Rates of adherence should be viewed cautiously due to use of self-report data, which may be influenced by recall and social desirability bias. The study design precludes determination of whether respondents were truly representative of the wider HCW population. However, associations within the data still provide useful insights.^9^ This study used cross-sectional data, limiting ability to infer causation. We gathered only limited sociodemographic data from participants, due to space constraints in the survey limiting the ability to determine representativeness of survey respondents to the NHS workforce. As we used quota sampling, it is misleading to calculate response rate (as once certain quotas (eg, based on age or sex) have been filled, respondents with these characteristics are prevented from completing the survey). Rates of completion and exclusion based on quality control procedures are typical for this method of data collection.

## Conclusion

Uptake of PPBs at the start of the COVID-19 pandemic among UK HCWs was suboptimal. Factors associated with adopting PPBs included having an organisational culture of adopting PPBs, adequate availability of resources and having a workplace design that facilitated adherence. Contrary to previous research, we found that participants with more regular contact with confirmed cases were less likely to fully adhere to PPE. This is likely a function of the greater frequency with which PPE is necessary with higher patient contact. Our results identify factors that could be targeted to increase uptake of PPBs among HCWs and highlight the need to support HCWs with frequent contact with COVID-19 cases to fully adhere.

## Data Availability

Data are available on reasonable request.
