# Supplementary material for: Adherence to protective measures among healthcare workers in the UK: a cross-sectional study
Source: Emerg Med J. 2021 Nov 30;39(2):100–5. doi: 10.1136/emermed-2021-211454 (PMC8788253; doi:10.1136/emermed-2021-211454)
Supplement: Supplementary data [file emermed-2021-211454supp001.pdf]

Supplementary materials. Non-significant results

Table of non-significant adjusted results (*p*≤0.002) for PPE analyses.

Participant characteristics	Level	Did not completely adhere to use of PPE n=166, n (%)	Completely adherent to use of PPE n=665, n (%)	Odds ratio (95% CI) for complete adherence to use of PPE	Adjusted odds ratio (95% CI)† for complete adherence to use of PPE
Sex	Male	48 (20.6)	185 (79.4)	Reference	Reference
	Female	118 (19.7)	480 (80.3)	1.06 (0.73 to 1.54)	1.16 (0.77 to 1.74)
Region of place of work ‡	North East	9 (23.1)	30 (76.9)	Reference	Reference
	North West	22 (21.6)	80 (78.4)	1.05 (0.43 to 2.54)	1.12 (0.44 to 2.86)
	Yorkshire and the Humber	11 (16.2)	57 (83.8)	1.51 (0.56 to 4.08)	1.77 (0.63 to 5.02)
	East Midlands	8 (14.0)	49 (86.0)	1.80 (0.62 to 5.22)	1.78 (0.58 to 5.45)
	West Midlands	13 (21.0)	49 (79.0)	1.08 (0.41 to 2.85)	0.98 (0.35 to 2.74)
	East of England	17 (32.1)	36 (67.9)	0.62 (0.24 to 1.59)	0.75 (0.28 to 2.04)
	London	15 (21.7)	54 (78.3)	1.01 (0.39 to 2.59)	0.89 (0.33 to 2.45)
	South East	27 (23.9)	86 (76.1)	0.93 (0.39 to 2.21)	0.86 (0.34 to 2.16)
	South West	13 (14.4)	77 (85.6)	1.73 (0.66 to 4.49)	1.71 (0.63 to 4.68)
	Wales	13 (25.0)	39 (75.0)	0.86 (0.32 to 2.28)	0.76 (0.27 to 2.17)
	Scotland	14 (13.0)	94 (87.0)	1.93 (0.75 to 4.92)	2.12 (0.80 to 5.67)
	Northern Ireland	5 (26.3)	14 (73.7)	0.86 (0.23 to 3.17)	0.99 (0.25 to 3.87)
Sector ‡	Private	43 (22.8)	146 (77.2)	Reference	Reference
	Public	123 (19.1)	520 (80.9)	1.24 (0.84 to 1.84)	1.10 (0.71 to 1.69)
Work setting ‡	Pharmacy, dentist, opticians, clinical commissioning group, mental health trust/service, community services, local authority, school, university, other	36 (15.8)	192 (84.2)	Reference	Reference
	NHS hospital, private hospital/ clinic, GP surgery/health centre, walk-in centre, ambulance trust/service, care home	130 (21.6)	473 (78.4)	0.69 (0.46 to 1.03)	0.70 (0.45 to 1.08)
Occupational group ‡	Allied health professionals/healthcare scientists/scientific and technical, public health/health improvement, commissioning managers/support staff, wider healthcare team (including admin & clerical, HR, finance, IT, facilities and maintenance), general management, other occupational group	48 (14.8)	277 (85.2)	Reference	Reference
	Medical and dental, ambulance, registered nurses and midwives, nursing or healthcare assistants, social care	75 (23.7)	242 (76.3)	0.56 (0.38 to 0.84)	0.74 (0.46 to 1.18)

Perceived risk of COVID-19 to me personally	5-point scale, 1=no risk at all to 5=major risk	N=164, M=3.32, SD=1.01	N=661, M=3.22, SD=0.95	0.90 (0.75 to 1.07)	0.95 (0.78 to 1.15)
Perceived risk of COVID-19 to people in the UK	5-point scale, 1=no risk at all to 5=major risk	N=165, M=3.76, SD=0.86	N=663, M=3.83, SD=0.82	1.10 (0.90 to 1.35)	1.18 (0.95 to 1.48)
Had, or currently have, COVID-19	Think have not had COVID-19 and do not have it now	109 (19.2)	459 (80.8)	Reference	Reference
	Think have had COVID-19 or have it now	42 (26.6)	116 (73.4)	0.66 (0.44 to 1.00)	0.73 (0.47 to 1.15)
Symptoms of COVID-19 in household ‡	None present	157 (19.9)	633 (80.1)	Reference	Reference
	Present	5 (17.9)	23 (82.1)	1.11 (0.42 to 2.94)	1.42 (0.51 to 3.96)
Perceived credibility of information from the NHS about PPE	Range 4 (lowest) to 20 (highest)	N=152, M=12.24, SD=2.25	N=592, M=12.97, SD=2.33	1.14 (1.06 to 1.24)*	1.13 (1.04 to 1.23)
If I was going to catch COVID-19, I would have done by now	5-point scale, 1=strongly disagree to 5=strongly agree	M=2.63, SD=1.18	M=2.53, SD=1.05	0.92 (0.78 to 1.07)	0.91 (0.77 to 1.08)
I am worried that if I don't take care, I might pass COVID-19 to my friends or family	5-point scale, 1=strongly disagree to 5=strongly agree	M=4.04, SD=0.96	M=3.98, SD=0.89	0.92 (0.76 to 1.12)	1.00 (0.81 to 1.22)
Perceived effectiveness of PPE	Range 3 (lowest) to 15 (highest)	M=9.38, SD=2.47	M=9.94, SD=2.37	1.10 (1.03 to 1.18)	1.07 (0.99 to 1.15)
If I don't wear the right PPE at work, my colleagues will notice	5-point scale, 1=strongly disagree to 5=strongly agree	M=3.82, SD=1.02	M=3.94, SD=0.98	1.12 (0.94 to 1.32)	1.27 (1.05 to 1.53)
If I don't wear the right PPE at work, I will probably catch COVID-19	5-point scale, 1=strongly disagree to 5=strongly agree	M=3.37, SD=0.98	M=3.35, SD=0.99	0.98 (0.82 to 1.16)	1.16 (0.96 to 1.40)
As far as I'm aware, there are people from my workplace who have been seriously ill with COVID-19	5-point scale, 1=strongly disagree to 5=strongly agree	M=3.48, SD=1.32	M=3.27, SD=1.39	0.89 (0.79 to 1.01)	0.93 (0.80 to 1.07)
COVID-19 would be a serious illness for me	5-point scale, 1=strongly disagree to 5=strongly agree	M=3.21, SD=1.05	M=3.38, SD=1.05	1.16 (0.98 to 1.36)	1.09 (0.91 to 1.31)
My line manager seems to take PPE and social distancing seriously	5-point scale, 1=strongly disagree to 5=strongly agree	M=3.64, SD=1.15	M=3.9, SD=1.05	1.23 (1.06 to 1.43)	1.24 (1.05 to 1.47)

*$p \leq 0.002$ (applying Bonferroni correction)

† Adjusting for sex, age, region of place of work, sector, work setting, and face-to-face contact with patients or service users.

‡ The number of valid cases in the table is different from the total count due to the use of weighted data and rounding errors.

For continuous variables, where N is the same as the column heading, it is not reported in individual cells.

Table of non-significant adjusted results (*p*≤0.002) for hand hygiene analyses.

Participant characteristics	Level	Did not wash their hands every time needed (n=268)	Washed their hands every time needed (n=564)	Odds ratio (95% CI) for washing hands every time needed	Adjusted odds ratio (95% CI)† for washing hands every time needed
Sex	Male	75 (32.2)	158 (67.8)	Reference	Reference
	Female	193 (32.3)	405 (67.7)	1.00 (0.72 to 1.38)	0.92 (0.66 to 1.29)
Age ‡	18 to 34 years	31 (35.6)	56 (64.4)	Reference	Reference
	35 to 44 years	54 (31.0)	120 (69.0)	1.24 (0.72 to 2.13)	1.35 (0.77 to 2.36)
	45 to 54 years	83 (32.7)	171 (67.3)	1.15 (0.69 to 1.92)	1.26 (0.74 to 2.13)
	55 years and over	99 (31.4)	216 (68.6)	1.22 (0.74 to 2.00)	1.29 (0.77 to 2.16)
Region of place of work ‡	North East	15 (38.5)	24 (61.5)	Reference	Reference
	North West	34 (33.3)	68 (66.7)	1.22 (0.57 to 2.62)	1.31 (0.60 to 2.84)
	Yorkshire and the Humber	25 (36.8)	43 (63.2)	1.06 (0.47 to 2.39)	1.10 (0.48 to 2.51)
	East Midlands	17 (29.8)	40 (70.2)	1.39 (0.59 to 3.29)	1.34 (0.56 to 3.19)
	West Midlands	21 (33.9)	41 (66.1)	1.16 (0.50 to 2.68)	1.21 (0.52 to 2.81)
	East of England	20 (37.7)	33 (62.3)	1.02 (0.44 to 2.41)	1.11 (0.47 to 2.63)
	London	18 (26.1)	51 (73.9)	1.74 (0.75 to 4.05)	1.78 (0.76 to 4.19)
	South East	22 (19.5)	91 (80.5)	2.45 (1.11 to 5.44)	2.53 (1.13 to 5.66)
	South West	27 (29.7)	64 (70.3)	1.43 (0.65 to 3.15)	1.51 (0.67 to 3.36)
	Wales	17 (32.7)	35 (67.3)	1.22 (0.51 to 2.90)	1.24 (0.51 to 2.98)
	Scotland	46 (42.2)	63 (57.8)	0.83 (0.39 to 1.76)	0.87 (0.41 to 1.87)
	Northern Ireland	8 (44.4)	10 (55.6)	0.82 (0.26 to 2.54)	0.86 (0.27 to 2.70)
Sector ‡	Private	77 (40.7)	112 (59.3)	Reference	Reference
	Public	191 (29.7)	452 (70.3)	1.62 (1.15 to 2.26)	1.50 (1.04 to 2.15)
Place of work ‡	Pharmacy, dentist, opticians, clinical commissioning group, mental health trust/service, community services, local authority, school, university, other	84 (36.8)	114 (63.2)	Reference	Reference
	NHS hospital, private hospital/ clinic, GP surgery/health centre, walk-in centre, ambulance trust/service, care home	184 (30.5)	420 (69.5)	1.33 (0.97 to 1.83)	1.23 (0.88 to 1.72)
Face-to-face contact with patients/service users ‡	No	34 (23.6)	110 (76.4)	Reference	Reference
	Yes, occasionally	41 (31.3)	90 (68.7)	0.67 (0.39 to 1.15)	0.71 (0.41 to 1.23)
	Yes, frequently	193 (34.6)	364 (65.4)	0.58 (0.38 to 0.89)	0.68 (0.44 to 1.05)
Occupational group ‡	Allied health professionals/healthcare scientists/scientific and technical, public health/health improvement, commissioning managers/support staff, wider healthcare team (including admin & clerical, HR, finance, IT, facilities and maintenance), general management, other occupational group	91 (27.9)	235 (72.1)	Reference	Reference

	Medical and dental, ambulance, registered nurses and midwives, nursing or healthcare assistants, social care	100 (31.5)	217 (68.5)	0.84 (0.60 to 1.17)	0.97 (0.66 to 1.43)
Frequency of contact with patients with COVID-19, or staff who worked closely with patients with COVID-19 ‡	I am never in contact myself with patients who have COVID-19 or anyone who has regular contact with patients who have COVID-19	88 (31.2)	194 (68.8)	Reference	Reference
	I am never in contact myself with patients who have COVID-19 but work closely with staff who have regular contact with patients who have COVID-19	32 (23.4)	105 (76.6)	1.49 (0.93 to 2.38)	1.23 (0.75 to 2.01)
	I am rarely in contact myself with patients who have COVID-19	48 (30.8)	108 (69.2)	1.01 (0.66 to 1.54)	0.96 (0.61 to 1.51)
	I am sometimes in contact myself with patients who have COVID-19	69 (40.6)	101 (59.4)	0.67 (0.45 to 0.99)	0.63 (0.40 to 0.99)
	I am often in contact myself with patients who have COVID-19	30 (34.9)	56 (65.1)	0.86 (0.52 to 1.44)	0.73 (0.41 to 1.29)
There are facilities at my workplace available that make it easy to wash my hands when I get to work	5-point scale, 1=strongly disagree to 5=strongly agree	M=4.14, SD=0.96	M=4.34, SD=0.85	1.27 (1.08 to 1.48)	1.29 (1.09 to 1.53)
I have received adequate training in my workplace for the purposes of health and safety during the COVID-19 pandemic (i.e., correct use of PPE and social distancing)	5-point scale, 1=strongly disagree to 5=strongly agree	M=3.50, SD=1.18	M=3.55, SD=1.18	1.03 (0.91 to 1.17)	1.07 (0.94 to 1.21)
In the past week, I have found a sink at my workplace where I couldn't wash my hands because it was broken	No	228 (31.5)	496 (68.5)	Reference	Reference
	Yes	16 (39.0)	25 (61.0)	0.72 (0.38 to 1.36)	0.71 (0.36 to 1.37)
In the past week, I have found a sink at my workplace with no soap or paper towels or a gel dispenser which was empty	No	182 (32.0)	386 (68.0)	Reference	Reference
	Yes	61 (31.1)	135 (68.9)	1.05 (0.74 to 1.49)	0.99 (0.68 to 1.44)
Perceived risk of COVID-19 to me personally	5-point scale, 1=no risk at all to 5=major risk	N=265, M=3.35, SD=0.99	N=560, M=3.19, SD=0.94	0.84 (0.72 to 0.98)	0.88 (0.75 to 1.03)
Perceived risk of COVID-19 to people in the UK	5-point scale, 1=no risk at all to 5=major risk	N=266, M=3.87, SD=0.83	N=563, M=3.79, SD=0.83	0.89 (0.75 to 1.07)	0.91 (0.76 to 1.10)
Had, or currently have, COVID-19 ‡	Think have not had COVID-19 and do not have it now	181 (31.9)	386 (68.1)	Reference	Reference
	Think have had COVID-19 or have it now	51 (32.5)	106 (67.5)	0.97 (0.66 to 1.41)	0.90 (0.61 to 1.34)
Symptoms of COVID-19 in household ‡	None present	254 (32.1)	537 (67.9)	Reference	Reference
	Present	9 (32.1)	19 (67.9)	1.00 (0.45 to 2.23)	0.97 (0.42 to 2.23)
Perceived credibility of information from the NHS about PPE	Range 4 (lowest) to 20 (highest)	N=247, M=12.67, SD=2.41	N=497, M=12.89, SD=2.29	1.04 (0.98 to 1.11)	1.05 (0.98 to 1.13)
It doesn't really matter what I do, I will probably catch COVID-19 anyway	5-point scale, 1=strongly disagree to 5=strongly agree	M=2.58, SD=0.97	M=2.54, SD=0.97	0.96 (0.82 to 1.11)	0.92 (0.79 to 1.08)
If I was going to catch COVID-19, I would have done by now	5-point scale, 1=strongly disagree to 5=strongly agree	M=2.58, SD=1.07	M=2.54, SD=1.08	0.96 (0.84 to 1.10)	0.97 (0.84 to 1.11)

I am worried that if I don't take care, I might pass COVID-19 to my friends or family	5-point scale, 1=strongly disagree to 5=strongly agree	M=3.91, SD=0.97	M=4.04, SD=0.87	1.17 (1.00 to 1.37)	1.18 (1.00 to 1.39)
As far as I'm aware, there are people from my workplace who have been seriously ill with COVID-19	5-point scale, 1=strongly disagree to 5=strongly agree	M=3.23, SD=1.39	M=3.35, SD=1.38	1.07 (0.96 to 1.19)	1.01 (0.90 to 1.14)
COVID-19 would be a serious illness for me	5-point scale, 1=strongly disagree to 5=strongly agree	M=3.38, SD=1.06	M=3.32, SD=1.04	0.95 (0.82 to 1.09)	0.97 (0.84 to 1.12)
I feel safe from COVID-19 at work	5-point scale, 1=strongly disagree to 5=strongly agree	M=3.02, SD=1.04	M=3.04, SD=1.06	1.02 (0.89 to 1.17)	1.02 (0.88 to 1.18)

*$p \leq 0.002$ (applying Bonferroni correction)

† Adjusting for sex, age, region of place of work, sector, work setting, and face-to-face contact with patients or service users.

‡ The number of valid cases in the table is different from the total count due to the use of weighted data and rounding errors.

For continuous variables, where N is the same as the column heading, it is not reported in individual cells.

Table of non-significant adjusted results ($p \leq 0.002$) for close contact in the workplace analyses.

Participant characteristics	Level	Were not in close contact with colleagues in the workplace (n=210)	Were in close contact with colleagues in the workplace (n=621)	Odds ratio (95% CI) for being in close contact with a colleague	Adjusted odds ratio (95% CI)† for being in close contact with a colleague
Sex	Male	63 (27.0)	170 (73.0)	Reference	Reference
	Female	147 (24.6)	451 (75.4)	1.13 (0.80 to 1.59)	0.94 (0.66 to 1.36)
Age	18 to 34 years	14 (17.2)	72 (82.8)	Reference	Reference
	35 to 44 years	35 (20.1)	139 (79.9)	0.86 (0.44 to 1.67)	0.97 (0.49 to 1.94)
	45 to 54 years	72 (28.2)	183 (71.8)	0.55 (0.30 to 1.02)	0.63 (0.34 to 1.20)
	55 years and over	88 (27.9)	227 (72.1)	0.55 (0.30 to 1.01)	0.66 (0.36 to 1.24)
Region of place of work ‡	North East	8 (20.5)	31 (79.5)	Reference	Reference
	North West	28 (27.5)	74 (72.5)	0.64 (0.26 to 1.59)	0.66 (0.26 to 1.67)
	Yorkshire and the Humber	21 (30.9)	47 (69.1)	0.53 (0.21 to 1.37)	0.47 (0.18 to 1.25)
	East Midlands	13 (22.8)	44 (77.2)	0.85 (0.31 to 2.34)	0.79 (0.28 to 2.24)
	West Midlands	16 (25.8)	46 (74.2)	0.71 (0.27 to 1.89)	0.81 (0.29 to 2.23)
	East of England	13 (24.5)	40 (75.5)	0.73 (0.27 to 2.00)	0.69 (0.25 to 1.96)
	London	18 (26.5)	50 (73.5)	0.66 (0.26 to 1.72)	0.71 (0.26 to 1.90)
	South East	25 (22.1)	88 (77.9)	0.84 (0.34 to 2.07)	0.87 (0.34 to 2.22)
	South West	21 (23.3)	69 (76.7)	0.79 (0.31 to 1.99)	0.85 (0.32 to 2.21)
	Wales	10 (19.2)	42 (80.8)	0.99 (0.35 to 2.81)	1.06 (0.36 to 3.11)
	Scotland	33 (30.6)	75 (69.4)	0.55 (0.23 to 1.34)	0.56 (0.22 to 1.41)
	Northern Ireland	4 (21.1)	15 (78.9)	0.97 (0.24 to 3.91)	0.90 (0.22 to 3.72)
Place of work ‡	Pharmacy, dentist, opticians, clinical commissioning group, mental health trust/service, community services, local authority, school, university, other	81 (35.4)	148 (64.6)	Reference	Reference
	NHS hospital, private hospital/ clinic, GP surgery/health centre, walk-in centre, ambulance trust/service, care home	130 (21.6)	473 (78.4)	1.99 (1.43 to 2.78)*	1.65 (1.16 to 2.35)
Face-to-face contact with patients/service users ‡	No	40 (28.0)	103 (72.0)	Reference	Reference
	Yes, occasionally	45 (34.4)	86 (65.6)	0.76 (0.45 to 1.26)	0.82 (0.48 to 1.39)
	Yes, frequently	125 (22.5)	431 (77.5)	1.35 (0.89 to 2.04)	1.68 (1.08 to 2.62)
Occupational group ‡	Allied health professionals/healthcare scientists/scientific and technical, public health/health improvement, commissioning managers/support staff, wider healthcare team (including admin & clerical, HR, finance, IT,	78 (24.0)	247 (76.0)	Reference	Reference

	facilities and maintenance), general management, other occupational group				
	Medical and dental, ambulance, registered nurses and midwives, nursing or healthcare assistants, social care	58 (18.3)	259 (81.7)	1.40 (0.96 to 2.05)	1.50 (0.96 to 2.35)
Frequency of contact with patients with COVID-19, or staff who worked closely with patients with COVID-19 ‡	I am never in contact myself with patients who have COVID-19 or anyone who has regular contact with patients who have COVID-19	100 (35.5)	182 (64.5)	Reference	Reference
	I am never in contact myself with patients who have COVID-19 but work closely with staff who have regular contact with patients who have COVID-19	31 (22.6)	106 (77.4)	1.90 (1.19 to 3.04)	1.68 (1.02 to 2.77)
	I am rarely in contact myself with patients who have COVID-19	35 (22.4)	121 (77.6)	1.90 (1.21 to 2.97)	1.59 (0.98 to 2.57)
	I am sometimes in contact myself with patients who have COVID-19	33 (19.4)	137 (80.6)	2.30 (1.46 to 3.61)*	1.61 (0.96 to 2.67)
	I am often in contact myself with patients who have COVID-19	11 (12.8)	75 (87.2)	3.74 (1.90 to 7.36)*	2.43 (1.17 to 5.05)
Perceived risk of COVID-19 to me personally	5-point scale, 1=no risk at all to 5=major risk	N=209, M=3.20, SD=0.90	N=616, M=3.25, SD=0.98	1.06 (0.90 to 1.25)	1.06 (0.89 to 1.27)
Perceived risk of COVID-19 to people in the UK	5-point scale, 1=no risk at all to 5=major risk	M=3.85, SD=0.82	N=618, M=3.80, SD=0.83	0.93 (0.77 to 1.13)	0.90 (0.74 to 1.10)
Had, or currently have, COVID-19	Think have not had COVID-19 and do not have it now	152 (26.8)	416 (73.2)	Reference	Reference
	Think have had COVID-19 or have it now	32 (20.3)	126 (79.7)	1.43 (0.93 to 2.21)	1.21 (0.77 to 1.90)
Symptoms of COVID-19 in household ‡	None present	202 (25.6)	588 (74.4)	Reference	Reference
	Present	3 (10.7)	25 (89.3)	2.77 (0.84 to 9.05)	2.25 (0.67 to 7.61)
It doesn't really matter what I do, I will probably catch COVID-19 anyway	5-point scale, 1=strongly disagree to 5=strongly agree	M=2.40, SD=0.98	M=2.61, SD=0.96	1.26 (1.06 to 1.49)	1.16 (0.97 to 1.39)
If I was going to catch COVID-19, I would have done by now	5-point scale, 1=strongly disagree to 5=strongly agree	M=2.46, SD=1.06	M=2.58, SD=1.08	1.12 (0.96 to 1.30)	1.17 (1.00 to 1.38)
I am worried that if I don't take care, I might pass COVID-19 to my friends or family	5-point scale, 1=strongly disagree to 5=strongly agree	M=3.97, SD=0.91	M=4.00, SD=0.91	1.04 (0.88 to 1.24)	0.97 (0.81 to 1.16)
COVID-19 would be a serious illness for me	5-point scale, 1=strongly disagree to 5=strongly agree	M=3.47, SD=0.96	M=3.30, SD=1.07	0.85 (0.73 to 0.99)	0.92 (0.78 to 1.09)
If I don't maintain social distancing at work, I will probably catch COVID-19	5-point scale, 1=strongly disagree to 5=strongly agree	M=3.28, SD=0.90	M=3.09, SD=0.91	0.79 (0.67 to 0.95)	0.79 (0.66 to 0.95)

*$p \leq 0.002$ (applying Bonferroni correction)

† Adjusting for sex, age, region of place of work, sector, work setting, and face-to-face contact with patients or service users.

‡ The number of valid cases in the table is different from the total count due to the use of weighted data and rounding errors.

BMJ Publishing Group Limited (BMJ) disclaims all liability and responsibility arising from any reliance placed on this supplemental material which has been supplied by the author(s)

For continuous variables, where N is the same as the column heading, it is not reported in individual cells.
