# Supplementary material for: Adherence to protective measures among healthcare workers in the UK: a cross-sectional study
Source: Emerg Med J. 2021 Nov 30;39(2):100–5. doi: 10.1136/emermed-2021-211454 (PMC8788253; doi:10.1136/emermed-2021-211454)
Supplement: Supplementary data [file emermed-2021-211454supp002.pdf]

**Supplementary materials. Recoding of variables**

We created a single binary variable indicating whether participants fully adhered to wearing PPE (mask, gloves, apron or gown and eye or face protection) or not. For these items, we coded people who wore items of PPE even when they did not need to as adherent.

We created a binary variable indicating whether participants had been in close contact with a colleague while at work.

We recoded whether people thought they had had COVID-19 or thought they had it now into, and presence of household symptoms into separate binary variables. Presence of symptoms was defined as a participant reporting that they had experienced cough, a high temperature / fever, or loss or change to their sense of smell or taste in the last seven days, or if a member of their household had experienced cough, a high temperature / fever, or loss or change to their sense of smell or taste in the last fourteen days. Under UK guidance at the time, either of these events should have resulted in the participant being required to not leave their home at all for a minimum of seven days.

We recoded perceived ease of maintaining physical distancing in different situations in the workplace and effectiveness of wearing PPE (face mask and gloves around patients, and face mask around colleagues) into separate continuous variables.

For all variables, unless stated otherwise, we coded answers of "don't know" as missing data.
