# Supplementary material for: Adherence to protective measures among healthcare workers in the UK: a cross-sectional study
Source: Emerg Med J. 2021 Nov 30;39(2):100–5. doi: 10.1136/emermed-2021-211454 (PMC8788253; doi:10.1136/emermed-2021-211454)
Supplement: Supplementary data [file emermed-2021-211454supp003.pdf]

Supplementary materials. Survey

Just to remind you – this survey is completely anonymous and there is no way that anyone will be able to identify which answers you provided or who you are. Please be honest in your answers!

#base: NHS staff only
[Q] {single} In the NHS how would you describe your occupational group? Hover over each for more detail.
<1> Allied Health Professionals / Healthcare Scientists / Scientific and Technical
<2> Medical and Dental
<3> Ambulance
<4> Public Health / Health Improvement
<5> Commissioning managers / support staff
<6> Registered Nurses and Midwives
<7> Nursing or Healthcare Assistants
<8> Social Care
<9> Wider Healthcare Team (inc. admin & clerical, HR, finance, IT, facilities and maintenance)
<10> Other General Management
<11> Other occupational group

[Q] {single} And which one of the following best describes your job role / position?
<1> Dental hygienist
<2> Dentist
<3> General practice doctor
<4> Hospital doctor
<5> Surgeon
<6> Medical related scientific services
<7> Pharmacist
<8> Radiographer
<9> Psychologist
<10> Mental health worker
<11> Occupational therapist
<12> Dietitian / Nutritionist
<13> Optometrist / Optician
<14> Orthoptist
<15> Physiotherapist
<16> Podiatrist
<17> Nurse
<18> Midwife
<19> Therapist
<20> Complementary medicine
<21> Ambulance Driver
<22> Paramedic
<23> Lab Technician
<24> Pharmacy Technician

<25> Scientist
<26> Analyst
<27> Medical Secretary
<28> Practice Manager
<29> Advice worker
<30> Youth worker
<31> Support Worker
<32> Counselling and psychotherapy
<33> Social Worker
<34> Care worker
<35> Fitness Instructor
<36> Health Care Assistant
<37> Health Visitor
<95 fixed> Other role

[Q] {single} Which, if any, of the following BEST describes where you work?
<1>NHS hospital
<2>Private hospital/ clinic
<3>GP surgery/ health centre
<4>Walk-in centre
<5>Ambulance trust/ service
<6>Pharmacy
<7>Dentist
<8>Opticians
<13> Clinical commissioning group
<14> Mental health trust / service
<9>Care home
<10>Community services
<11>Local Authority
<101>School
<102>University
<12>Other [Organisation_type_other] {open}

[Q] {single} Do you have face-to-face contact with patients / service users as part of your job?
<1>Yes, frequently
<2>Yes, occasionally
<3>No

Q3. What is your occupational group? (If Non-clinical for Q2 – single code)

1. Administrative and Clerical
2. Catering Services
3. Chaplaincy
4. Clinical Support
5. Domestic Services
6. Estates Services
7. Finance
8. Healthcare Scientists (laboratory, technician)
9. Human Resources

10. IT Support
11. Management
12. Research / Academic

Q1. To what extent, if at all, do you think the coronavirus (COVID-19) poses a risk to:

SCALE:

1. Major risk
2. Significant risk
3. Moderate risk
4. Minor risk
5. No risk at all
6. Don’t know

STATEMENTS:

- People in the UK?
- You personally?

Q2. In the past **seven days**, which, if any, of the following symptoms have you experienced? Please select all that apply. [Multicode]

1. A new, continuous cough
2. High temperature / fever
3. Shortness of breath / difficulties breathing
4. Runny or blocked nose
5. Aches and pains
6. Chest pain
7. Chills / shivering
8. Sore throat
9. Diarrhoea
10. Headache
11. Stomach-ache
12. Feeling tired or having low energy
13. Loss or change to your sense of smell or taste
14. None of these
15. Don’t know

Q3. In the past **<u>fourteen days</u>**, which, if any, of the following symptoms has **<u>someone else in your household</u>** experienced? Please select all that apply. [Multicode]

1. A new, continuous cough
2. High temperature / fever
3. Shortness of breath / difficulty breathing
4. Runny or blocked nose
5. Aches and pains
6. Chest pain
7. Chills / shivering
8. Sore throat
9. Diarrhoea

10. Headache
11. Stomach-ache
12. Feeling tired or having low energy
13. Loss or change to your sense of smell or taste
14. None of these
15. Don’t know

Q4. In the past **twenty-four hours**, how many times, if at all, have you left your home for each of the following reasons? Please type your answers in the boxes below. [Record number 0-50 for each item]

1. To go to the shops, mainly for groceries, toiletries or medicine
2. To go to the shops, mainly for other items
3. For exercise
4. To go to work
5. To help someone else (e.g. delivered medicine or done their shopping for them)
6. To meet friends or family who do not live with you
7. Other
8. Not applicable – I have not left my home in the past 24 hours

Q5. In the past **twenty-four hours**, have you been to visit a friend or family member who does not live with you, and been inside their home?

1. Yes
2. No

Q7. Which of the following best describes whether or not you have had, or currently have, COVID-19? [Single code]

1. I have definitely had it, or definitely have it now
2. I have probably had it, or probably have it now
3. I have probably not had it, and probably don’t have it now
4. I have definitely not had it, and definitely don’t have it now
5. Don’t know

Just to remind you once again – this survey is completely anonymous and there is no way that anyone will be able to identify which answers you provided or who you are. We would very much appreciate you providing truthful responses.

Q8. Which of the following best applies to you, at work? [single code]

1. I am never in contact myself with patients who have COVID-19 or anyone who has regular contact with patients who have COVID-19
2. I am never in contact myself with patients who have COVID-19 but work closely with staff who have regular contact with patients who have COVID-19
3. I am rarely in contact myself with patients who have COVID-19
4. I am sometimes in contact myself with patients who have COVID-19
5. I am often in contact myself with patients who have COVID-19

Q9. Thinking about the most recent time you were at work, which of the following best applied? In this question, by ‘mask’ we mean a standard face mask, **not** a N95/FFP2/FPP3 mask. [single code]

1. I was supposed to wear a mask, and I managed to wear it every time I was meant to
2. I was supposed to wear a mask, but I didn’t manage to wear it every time I was meant to
3. I wasn’t supposed to wear a mask, but I wore one anyway
4. I wasn’t supposed to wear a mask, and I did not wear one
5. Not applicable – I have not physically been to work over the last six weeks

#base: excluding those who have not physically been to work over the last six weeks

Q10. Thinking about the most recent time you were at work, which of the following best applied? [single code]

1. I was supposed to wear gloves, and I managed to wear them every time I was meant to
2. I was supposed to wear gloves, but I didn’t manage to wear them every time I was meant to
3. I wasn’t supposed to wear gloves, but I wore them anyway
4. I wasn’t supposed to wear gloves, and I did not wear them

#base: excluding those who have not physically been to work over the last six weeks

Q11. Thinking about the most recent time you were at work, which of the following best applied? [single code]

1. I was supposed to wear an apron or gown, and I managed to wear one every time I was meant to
2. I was supposed to wear an apron or gown, but I didn’t manage to wear one every time I was meant to
3. I wasn’t supposed to wear an apron or gown, but I wore one anyway
4. I wasn’t supposed to wear an apron or gown, and I did not wear one

#base: excluding those who have not physically been to work over the last six weeks

Q12. Thinking about the most recent time you were at work, which of the following best applied? [single code]

1. I was supposed to wear face or eye protection (other than a mask), and I managed to wear it every time I was meant to
2. I was supposed to wear face or eye protection (other than a mask), but I didn’t manage to wear it every time I was meant to

3. I wasn't supposed to wear face or eye protection (other than a mask), but I wore it anyway
4. I wasn't supposed to wear face or eye protection (other than a mask), and I did not wear it

#base: excluding those who have not physically been to work over the last six weeks

Q13. Thinking about the most recent time you were at work which, if any, of the following applied? [multicode]

1. I managed to wash my hands with soap and water for 20 seconds or apply hand gel as soon as I got to work
2. I managed to wash my hands with soap and water for 20 seconds or apply hand gel every time I needed to while I was at work
3. I managed to wash my hands with soap and water for 20 seconds or apply hand gel before eating at work
4. I managed to wash my hands with soap and water for 20 seconds or apply hand gel as soon as I got home
5. I had a shower before leaving work or as soon as I got home
6. None of these applied

#base: excluding those who have not physically been to work over the last six weeks

Q14. We are interested in how easy or difficult it is for people to maintain social distancing at work. For these questions, we are interested in whether you were near to someone else who works in the health sector. By near, we mean **within 2 meters for 15 minutes or more**. Thinking about the most recent time you were at work, which of these, if any applied? [Multicode]

1. I was near to someone else during a team meeting
2. I was near to someone else on a ward/unit/clinical area when not wearing PPE
3. I was near to someone else in a break room, cafe or canteen
4. I near to someone else in a corridor
5. I had direct physical contact with someone else (e.g. a hug, handshake, pat on the back)
6. None of these applied to me

#base: excluding those who have not physically been to work over the last six weeks

Q15. For each of the following statements, please indicate the extent to which you agree or disagree.

SCALE:

1. Strongly agree
2. Agree

3. Neither agree nor disagree
4. Disagree
5. Strongly disagree

STATEMENTS: [Randomise order - single code for each]

- It doesn't really matter what I do, I will probably catch COVID-19 anyway
- If I was going to catch COVID-19, I would have done by now
- I am worried that if I don't take care, I might pass COVID-19 to my friends or family
- Wearing an ordinary surgical face mask around patients is an effective way to protect against COVID-19
- Wearing gloves around patients is an effective way to protect against COVID-19
- Social distancing around colleagues at work is an effective way to protect against COVID-19
- Wearing an ordinary surgical face mask around colleagues is an effective way to protect myself against COVID-19
- If I don't wear the right PPE at work, my colleagues will notice
- If I don't maintain social distancing at work, my colleagues will notice
- If I don't wear the right PPE at work, I will probably catch COVID-19
- If I don't maintain social distancing at work, I will probably catch COVID-19
- As far as I'm aware, there are people from my workplace who have been seriously ill with COVID-19
- I am angry about the way PPE has been given out to me or other healthcare workers
- COVID-19 would be a serious illness for me
- I feel safe from COVID-19 at home
- I feel safe from COVID-19 at work
- I feel safe from COVID-19 when out and about
- There is no point bothering with PPE around colleagues or social distancing if you already have a lot of contact with COVID-19 patients
- My colleagues seem to take PPE and social distancing seriously
- My line manager seems to take PPE and social distancing seriously
- Wearing PPE makes it hard for me to do my job properly

#base: excluding those who have not physically been to work over the last six weeks

Q16. For each of the following statements, please indicate the extent to which you agree or disagree. [Randomise order - single code for each – Strongly agree, agree, neither agree nor disagree, disagree, strongly disagree]

1. There are facilities at my workplace available that make it easy to wash my hands when I get to work
2. My workplace has clear markings which help me stay 2 meters away from other people
3. I have received adequate training in my workplace for the purposes of health and safety during the COVID-19 pandemic (i.e., correct use of PPE and social distancing)
4. I am given all the correct personal protective equipment that I need to do my job safely

5. I have enough information about which personal protective equipment to use and when to use it
6. The way my workplace is designed makes it easy for me to stay 2 meters away from other people

#base: excluding those who have not physically been to work over the last six weeks

Q16a. For the following two statements, please indicate whether this has occurred at your workplace in the past week. [Yes, this has occurred; No, this has not occurred; Don’t know / Not applicable – I have not been to my workplace in the past week]

1. In the past week, I have found a sink at my workplace where I couldn’t wash my hands because it was broken
2. In the past week, I have found a sink at my workplace with no soap or paper towels or a gel dispenser which was empty

#base: excluding those who have not physically been to work over the last six weeks

Q17To what extent do you think it is easy or difficult to keep 2m (3 steps) away from other people in the following situations at your workplace?

SCALE:

1. Very easy
2. Somewhat easy
3. Neither easy nor difficult
4. Somewhat difficult
5. Very difficult
6. Not applicable

STATEMENTS:

- Eating in canteens
- During rest breaks
- When saying hello or goodbye to colleagues
- When carrying out work that **does not** involve patient contact
- When carrying out work that **does** involve patient contact
- When moving from area to area (e.g. in corridors)

Q18. For each of the following statements, please indicate the extent to which you agree or disagree.

SCALE:

1. Strongly agree
2. Agree
3. Neither agree nor disagree
4. Disagree
5. Disagree strongly

6. Don't know

STATEMENTS:

- Information from the NHS about PPE can be trusted
- Information from the NHS about PPE is accurate
- Information from the NHS about PPE tells the whole story
- Information from the NHS about PPE is biased or one-sided

Q19. In which one of the following ways would you most **prefer** to get updates related to PPE (e.g. how to use, availability etc.)? [single code]

1. Team meetings at your workplace
2. Email circulars from your work
3. Your line manager
4. The NHS website
5. The PHE website
6. Your NHS trust / work website
7. Posters or leaflets at work
8. From the infection prevention and control team
9. Other (please write in)
